# NUP85 alleviates lipid metabolism and inflammation by regulating PI3K/AKT signaling pathway in nonalcoholic fatty liver disease

**DOI:** 10.7150/ijbs.92337

**Published:** 2024-03-25

**Authors:** Yin-cui Wu, Qi Yan, Si-qing Yue, Lin-xin Pan, Da-shuai Yang, Liang-song Tao, Ze-yuan Wei, Fan Rong, Cheng Qian, Meng-qi Han, Fu-cheng Zuo, Jun-fa Yang, Jia-jia Xu, Zheng-rong Shi, Jian Du, Zhao-lin Chen, Tao Xu

**Affiliations:** 1 Inflammation and Immune Mediated Diseases Laboratory of Anhui Province, Anhui Institute of Innovative Drugs, School of Pharmacy, Anhui Medical University, Hefei, 230032, China.; 2 Institute for Liver Diseases of Anhui Medical University, Hefei 230032, China.; 3 School of Basic Medical Sciences, Anhui Medical University, Hefei 230032, China.; 4 College of life sciences, Anhui Medical University, Hefei 230032, China.; 5 Research and Experiment center, Anhui Medical University, Hefei 230032, China.; 6 Department of Hepatobiliary Surgery, The First Affiliated Hospital of Chongqing Medical University, Chongqing, China.; 7 Department of Pharmacy, The First Affiliated Hospital of USTC, Division of Life Sciences and Medicine, University of Science and Technology of China, Anhui Provincial Hospital, Hefei, Anhui, 230001, China.; 8 Anhui Provincial Key Laboratory of Precision Pharmaceutical Preparations and Clinical Pharmacy, Hefei, Anhui, 230001, China.

**Keywords:** NUP85, Nonalcoholic fatty liver disease, PI3K/AKT, CCR2, ISRIB

## Abstract

Nonalcoholic fatty liver disease (NAFLD) is one of the common causes of chronic liver disease in the world. The problem of NAFLD had become increasingly prominent. However, its pathogenesis is still indistinct. As we all know, NAFLD begins with the accumulation of triglyceride (TG), leading to fatty degeneration, inflammation and other liver tissues damage. Notably, structure of nucleoporin 85 (NUP85) is related to lipid metabolism and inflammation of liver diseases. In this study, the results of researches indicated that NUP85 played a critical role in NAFLD. Firstly, the expression level of NUP85 in methionine-choline-deficient (MCD)-induced mice increased distinctly, as well as the levels of fat disorder and inflammation. On the contrary, knockdown of NUP85 had the opposite effects. *In vitro*, AML-12 cells were stimulated with 2 mm free fatty acids (FFA) for 24 h. Results also proved that NUP85 significantly increased in model group, and increased lipid accumulation and inflammation level. Besides, NUP85 protein could interact with C-C motif chemokine receptor 2 (CCR2). Furthermore, when NUP85 protein expressed at an extremely low level, the expression level of CCR2 protein also decreased, accompanied with an inhibition of phosphorylation of phosphoinositol-3 kinase (PI3K)-protein kinase B (AKT) signaling pathway. What is more, trans isomer (ISRIB), a targeted inhibitor of NUP85, could alleviate NAFLD. In summary, our findings suggested that NUP85 functions as an important regulator in NAFLD through modulation of CCR2.

## 1. Introduction

NAFLD is a heterogeneous pathological state of liver, which is caused by immoderate infiltration of fat in liver cells under other factors except alcohol 1. The disease progresses from nonalcoholic fatty liver (NAFL) to nonalcoholic steatohepatitis (NASH), and finally to NASH-related cirrhosis 2. Recently, the prevalence rate of NAFLD has risen considerably, impacting about 25% population in the world 3, 4, 5. Studies have indicated that various factors, including inflammation and lipid accumulation, can induce diversified stress responses such as metabolic stress, oxidative stress and endoplasmic reticulum stress in NAFLD 6. When the stress response was out of balance, damaged liver cells released signals to repair pathological changes, exacerbating and amplifying the lipid accumulation and inflammation in the liver. Therefore, the inhibition of steatosis and inflammation had become the key to prevent the deterioration of NAFLD 7. Studies have brought new ideas for the treatment of NAFLD by exploring the formation mechanism and searching for drug targets. Unfortunately, existing drugs for NAFLD still could not meet the increasing clinical demand 8. Therefore, it is of great practical significance to search a new target and drug related to new target for the treatment of NAFLD.

Nuclear pore complexes (NPCs) are large macromolecular complexes embedded in the nuclear membrane that control the bidirectional molecular transport channels between cytoplasm and nucleus 9, 10. NPCs are composed of a variety of nucleoporins (NUPs), including NUP160, NUP155, and NUP85 11, 12. More and more evidences have confirmed that NUPs are related to lipid metabolism and inflammation of liver diseases 13. For example, NUP155 was a part of the hepatocellular carcinoma (HCC) regulatory network and had been shown to inhibit the progression of HCC 14. NUP85 had been found to bind to chemokine receptors and mediate migration of leukocyte and monocyte cells 9. In the human protein map, NUP85 is widely expressed in various organs, especially in the liver 15. Furthermore, it was determined that NUP85 is extensively expressed in multiple, particularly liver cells. Overly, it is highly possible that NUP85 could play an essential role in lipid accumulation and inflammation in NAFLD.

However, the potential role of NUP85 and its mechanism of action in NAFLD remain unknown. In this study, we focus on the NUP85 and lipid accumulation and inflammation in NAFLD. NUP85 interacted with CCR2 to regulate the PI3K/AKT signaling pathway, thus affecting lipid homeostasis and inflammation. In addition, ISRIB, as a small molecule drug targeting NUP85, has been preliminarily studied for its role in NAFLD.

## Materials and methods

### Reagent

AML-12 cells are preserved in the School of Pharmacy, Anhui Medical University. Anti-NUP85 was bought from Santa Cruz Biotechnology (CA, USA). Anti-CCR2 was bought from Abcam (Cambridge, UK). Anti-phosphorylated protein kinase B (p-AKT), anti-AKT, anti-phosphorylated phosphatidylinositol-3 kinase (p-PI3K) and anti-PI3K were bought from Immunoway (Suzhou, China). Anti-β-actin, anti-acyl-CoAoxidase-1 (ACOX-1) and anti-interleukin-1beta (IL-1β) were got from Proteintech (Wuhan, China). Anti-sterol regulatory element binding protein-1 (SREBP-1C) was purchased from Affinity Biotechnology (Liyang, China). Anti-peroxisome proliferator-activated receptor alpha (PPAR-α), anti-interleukin-6 (IL-6) and anti-tumor necrosis factor-alpha (TNF-α) were got from Wanleibio (Shengyang, China). IL-1β, IL-6 and TNF-α ELISA kits were purchased from ELK Biotechnology CO., Ltd. (Wuhan, China). Oil red O staining kit was purchased from Solabio (Beijing, China). LY294002 and ISRIB were bought from Med Chem Express (Shanghai, China). Alanine aminotransferase (ALT), TG and aspartate aminotransferase (AST) kits were bought from Mindray (Shenzhen, China).

### Human liver samples

Fifty human liver tissues samples (44 NAFLD patients and 6 healthy subjects) were collected from the First Affiliated Hospital of the University of Science and Technology of China (USTC) and the First Affiliated Hospital of Chongqing Medical University. The diagnosis of each case was confirmed by pathologists based on WHO classification. Normal liver samples were the distal para-hemangioma normal tissues from patients undergoing surgical resection for hepatic hemangioma 16. Patients with NAFLD were diagnosed as steatosis with or without lobular inflammation and ballooning 17. This present study was approved by the Ethical Committee of the First Affiliated Hospital of USTC (No: 2023KY-030) and Chongqing Medical University (No: 2019-021) and all patients signed an informed consent. The baseline characteristics are detailed in Supplementary Table 1.

### Animal experiments

The experiment used 6-8-weeks male C57BL/6J mice. Mice were purchased from Gempharmatech Co., Ltd. All animal testing procedures have been approved by the ethical guidelines and reviewed and implemented in accordance with the standards of the Experimental Animal Ethics Committee of the First Affiliated Hospital of USTC (2023-N (A) -78). The mice were placed in a clean environment (22-24 °C, 55-60% humidity) on a light/dark cycle for 12 h. Methionine-choline-deficient (MCD) diet (TP3005G) and methionine-choline supplementation (MCS) diet (TP3005GS) were produced by Nantong Trophy Feed Technology Co., Ltd (Nantong, China). Adeno-associated virus (AAV8-shRNA-NUP85) was built by General biology Co., Ltd (Chuzhou, China). Sixty mice were randomly allotted among six groups subjected to different feeding plan (n=10). The six groups are MCS group, MCD group, AAV8-empty treated MCS-fed group, AAV8-empty treated MCD-fed group, AAV8-shRNA-NUP85 treated MCS-fed group and AAV8-shRNA-NUP85 treated MCD-fed group. The MCS group was fed with MCS diet and the MCD group was fed with MCD diet. AAV8-empty or AAV8-shRNA-NUP85 plasmid was injected into the mice through the tail vein. Besides, in order to verify the effect of ISRIB on NAFLD, other thirty mice were randomly divided into three groups (n=10). The three groups are MCS group, MCD group and MCD+ISRIB group. ISRIB was injected intraperitoneally (ip) into the mice at a concentration of 2.5 mg/kg every day for four weeks. All mice had adequate diet and continued to be fed for four weeks, during which their weight was measured weekly. After four weeks, the mice were all sacrificed, the mice serum and the liver tissues were gathered for subsequent study.

### Cell culture

AML-12 cells were cultured in a 37 °C incubator containing 5% CO_2_. The cell density inoculated in the six-well plate was approximately 1×10^6^. AML-12 cells were stimulated with FFA for 24 h. FFA was composed of 0.5 mmol. L ^-1^ oleic acid (OA) and 0.25 mmol. L^-1^ palmitic acid (PA).

### Oil Red O Staining

Frozen slices of liver tissues were used for Oil Red O Staining. The method of Oil Red O staining was described in the literature 18. For cell experiments, all the experimental steps were carried out according to the instructions of Oil Red O staining kit (Solarbio Technology).

### Cell proliferation assay

In 96-well plates, about 5000 cells were inoculated into each well, and cultured in an incubator with 100 μl medium. The cells were then cultured with different concentrations of FFA for 24 h. 90 μl medium and 10 μl CCK-8 solution were added to each well, and all cells were cultured at 37 ℃ for 1.5 h in darkness. Finally, the absorbance was measured at 450 nm using a microplate reader.

### Western blotting

Immunoblotting procedures could be found in reference 19. The first antibody included NUP85 (1:1000), CCR2 (1:1000), TNF-α (1:1000), IL-6 (1:1000), IL-1β (1:1000), PPAR-α (1:1000), ACOX-1 (1:1000), SREBP-1C (1:1000) and β-actin (1:3000). Finally, quantitative analysis was carried out with ImageJ software (version 1.8.0; National Institutes of Health).

### Total RNA extraction and real-time fluorescence quantitative PCR(RT-qPCR)

Total RNA was extracted from frozen liver tissues and cultured cells. After reverse transcription, quantitative and RT-qPCR were performed using Bio-Rad iQ SYBR Green Supermix and Opticon2 (Bio-Rad, Hercules, CA) according to the manufacturer's instructions. The folding changes of targeted genes mRNA level were related to the GAPDH.

### Immunohistochemistry (IHC)

Mice liver tissues were obtained and immediately soaked it in general tissue fixative solution for 24 h (5 μm) 20. The tissue was then photographed using an optical microscope.

### Immunofluorescence (IF)

The co-localization of NUP85 and Albumin in paraffin section of NAFLD was higher by immunofluorescent staining. After fixation, the cells were sealed with goat serum and incubated with primary antibodies (anti-NUP85 and anti-Albumin) in a wet chamber at 4 °C overnight. AML-12 cells were incubated with the anti-NUP85 (1:100) and anti-CCR2 (1:100) for 12 h, and then cultured for 2 h with suitable secondary antibodies. Eventually, the nucleus was stained with DAPI. All images were taken by fluorescent microscope (leica, Wetzlar, Germany).

### Haematoxylin eosin (HE) staining

Mice liver tissues were stored in general tissue fixation solution, paraffin-embedded, sliced (5 μm thick), dyed with neutral glue, placed under covered glass, and then observed under a fluorescence-inverted microscope 21.

### TUNEL staining

The liver tissues were encased in paraffin, the sections were deparaffinized. The antigen was extracted with a working solution of protease K and then treated by osmosis. After equilibrating for 10 min at room temperature, TUNEL reaction solution was added for incubation. After 1 h, the nucleus was re-stained with DAPI, and finally the film was taken 22.

### Co Immunoprecipitation (CO-IP) test

Co-IP was performed using the protein A/G beads kit (Biolinkedin, Shanghai, China) in AML-12 cells 23. Cells were lysed in a pre-cooled Western IP buffer. Anti-NUP85 was treated with protein A/G magnetic beads for 2 h at 4 °C, followed by incubating with total protein lysate overnight.

### Cellular thermal shift assay (CETSA)

The cells were divided into two groups, one stimulated with (80 um) ISRIB for 24 h and the other not. Next, cells were collected and resuspended with PBS to 1×10^9^ cells.ml^-1^. The cell suspension was evenly mixed, divided into 6 equal parts and heated at different temperatures for 10 min (37, 45, 50, 55, 60 and 65 °C). In the end, cell lysates were extracted by centrifugation at 12,000×g for 30 min 24. The protein level of NUP85 was assessed by Western blotting.

### Biochemical analysis

After the serum was collected, it was kept at room temperature for 40 minutes, and then centrifuged at 3000 rpm for 15 min at 4 °C to obtain serum 25. Serum AST, ALT and TG levels were measured according to the guidelines specified in the kit instructions.

### Hepatic parenchymatous cells Isolation

The separation of liver cells is described in reference 26.

### Statistical analysis

Statistical analyses were conducted in a rigorous manner, adhering to stringent criteria for inclusion. All experiments were repeated independently at least three times. All data are expressed as the mean ± standard error of mean (SEM). To ensure the normal distribution of data, the Kolmogorov-Smirnov test was used. Comparative statistical analyses between two groups were executed using the independent t-test. In scenarios involving multiple comparisons, a one-way analysis of variance (ANOVA) was initially employed, followed by Tukey's multiple comparison post hoc tests for further exploration. The statistical software utilized for these analyses was GraphPad Prism 8.0.2 (GraphPad Software Inc., San Diego, CA, USA). For one-way ANOVA, post-hoc tests were exclusively conducted if the achieved F-value demonstrated P < 0.05, and homogeneity of variance was confirmed. Statistical significance was established at *p < 0.05, **p < 0.01 and ***p < 0.001.

## Results

### The expression level of NUP85 is increased in the liver of NAFLD patients and model mice

**Figure 1:**
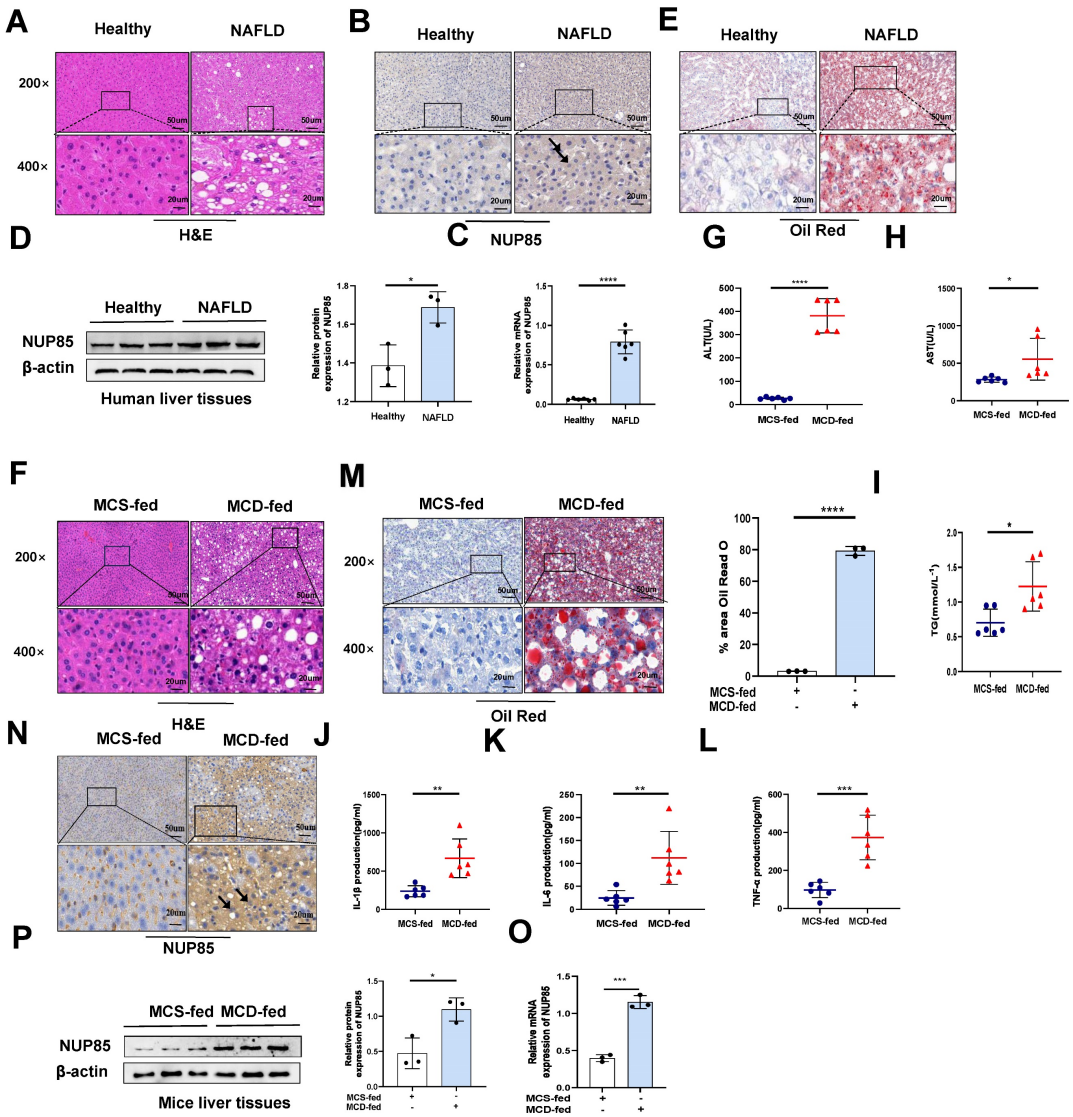
** The expression level of NUP85 is increased in the liver of NAFLD patients and model mice.** A. Human liver tissues stained with HE. B. Expression levels of NUP85 in liver of healthy people and NAFLD patients. C-D. RT-qPCR and Western blotting were used to detect the expression level of NUP85. E. Oil Red O staining of NAFLD patients. The percentage of lipid area in liver sections was detected by Oil red O staining. F. HE staining of mice liver tissues. G-I. The levels of ALT, AST and TG in serum. M. Oil Red O staining of mice tissues. The percentage of lipid area in liver sections was detected by Oil red O staining. J-L. Levels of serum IL-1β, IL-6 and TNF-α. N. Immunohistochemical analysis of NUP85 in MCS group and MCD group. O-P. The expression level of NUP85 was detected by RT-qPCR and Western blotting. Measurement metrics are shown in the figure. All experimental results of this study were replicated at least three times. **p<0.01, ***p<0.001 compared with the pair group.

Firstly, immunohistochemical experiments (Figure 1B), RT-qPCR (Figure 1C) and Western blotting (Figure 1D) were used to investigate the change of NUP85 expression level in the liver of NAFLD patients. The results indicated that the expression levels of NUP85 were greatly increased in the liver tissues of NAFLD patients. Based on histopathological examination, normal hepatic lobules were neatly formed around the central vein and had no obvious steatosis in the healthy group. In contrast, liver tissues in patients with NAFLD are characterized by fat vacuoles, disorganization of the liver cell line, expansion of the cell space, and infiltration of inflammatory cells (Figure 1A). Additionally, Oil Red O staining showed that the liver steatosis in NAFLD patients was more serious (Figure 1E). To investigate the effects of NUP85 in MCD-induced C57BL/6J mice, MCD model was established (Figure S1A). In line with earlier studies, the mice in the MCD group gradually lost weight during modeling (Figure S1B-1C). The liver of mice fed the MCD diet showed steatosis and damage. The liver of mice fed the MCD diet looked more yellow than that of mice fed with MCS diet (Figure S1D). We conducted a histopathological study to investigate the effects of MCD diet on mice. The degree of liver injury of fatty liver in mice fed with MCD diet was evaluated by HE staining (Figure 1F). Healthy liver lobules were formed neatly around the central vein in MCS-fed mice, and no significant steatosis was observed. In contrast, liver tissues of MCD-fed mice showed vacuoles and infiltration of inflammatory cells. The levels of serum ALT, AST and TG in MCD group were greatly increased (Figure 1G-1I). Compared with the MCS group, the levels of IL-1β, IL-6, and TNF-α in serum were significantly raised in the MCD group (Figure 1J-1L). Additionally, Oil Red O staining showed that the liver steatosis in MCD group was more serious (Figure 1M). The results of TUNEL staining revealed that the MCD group led to an increase in the number of apoptotic liver cells (Figure S1E). Besides, immunohistochemical staining (Figure 1N), RT-qPCR (Figure 1O) and Western blotting (Figure 1P) suggested that NUP85 expression level was elevated in the MCD group mice compared to the MCS group mice. Notably, immunofluorescence double staining showed that NUP85 and Albumin were co-localized in liver tissues (Figure S1F), which indicated that NUP85 is expressed in liver cells. These results confirmed that NUP85 levels in NAFLD patients and MCD-fed mice were increased, which may aggravate liver injury.

### NUP85 silencing alleviates inflammation and lipid accumulation in primary liver cells

**Figure 2:**
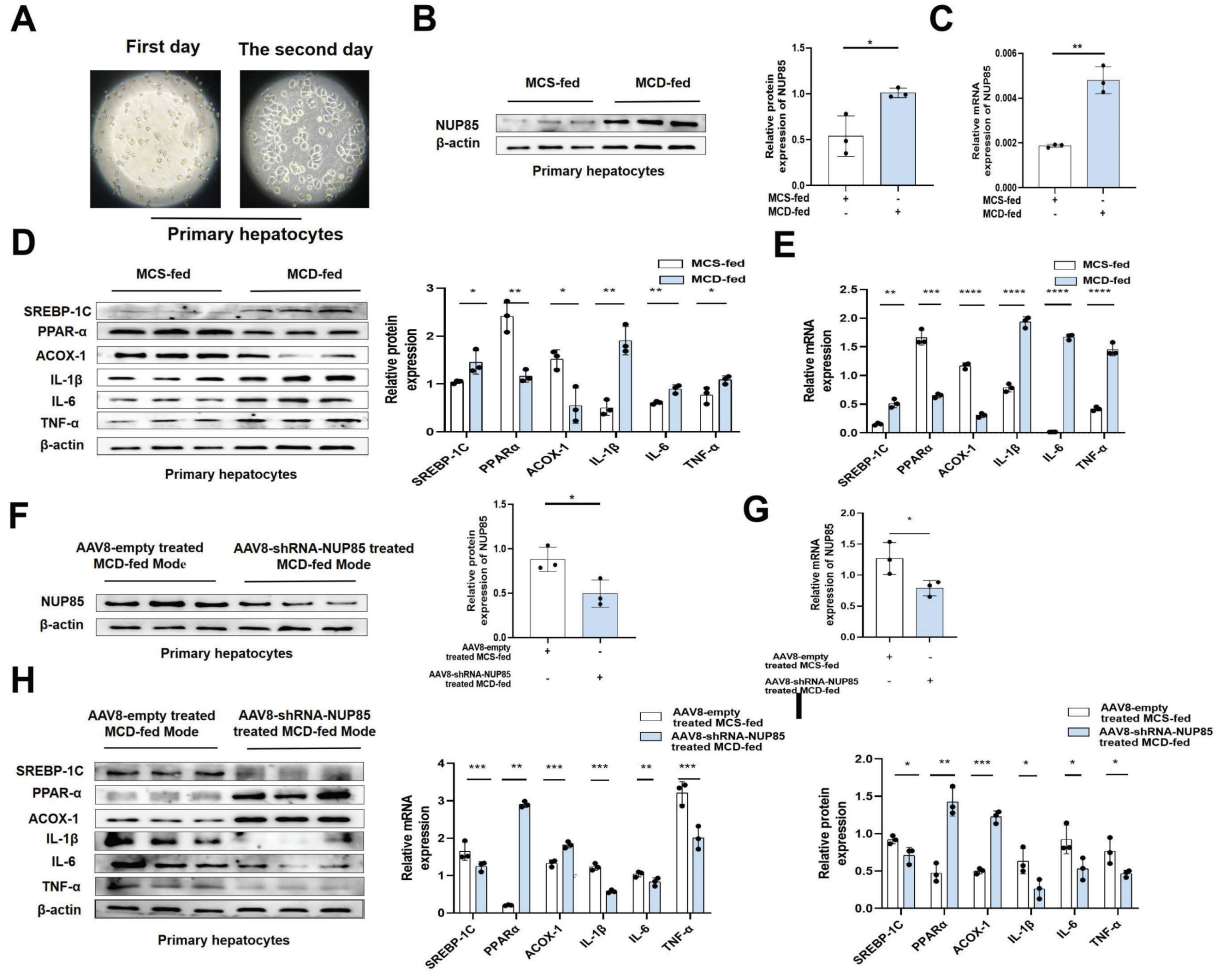
** NUP85 silencing alleviates inflammation and lipid accumulation in primary liver cells.** A. Image of extracted primary liver cells. B-E. The mRNA and protein expression levels of NUP85, SREBP-1C, PPAR-α, ACOX-1, IL-1β, TNF-α and IL-6 in the MCS group and MCD group. F-I. The mRNA and protein expression levels of NUP85, SREBP-1C, PPAR-α, ACOX-1, IL-1β, IL-6 and TNF-α in control NUP85 knockdown primary liver cells and NUP85 knockdown primary liver cells. All experimental results of this study were replicated at least three times. **p<0.01, ***p<0.001 compared with the control group.

Primary liver cells were extracted from liver tissues of model mice (Figure 2A), and used for subsequent research. The results showed that the expression levels of NUP85, SREBP-1C, IL-1β, TNF-α and IL-6 were increased in the MCD group, while PPAR-α and ACOX-1 expression levels were decreased (Figure 2B-2E). On the contrary, the expression levels of NUP85, SREBP-1C, IL-1β, IL-6 and TNF-α were down-regulated in the NUP85 knockdown group. While the expression levels of PPAR-α and ACOX-1 were up-regulated in the NUP85 knockdown group (Figure 2F-2I). To sum up, these results confirmed that the key role of NUP85 in liver was partly regulated by reducing lipid accumulation and inflammation. Overall, NUP85 silencing alleviates inflammation and lipid accumulation in primary liver cells.

### The expression level of NUP85 is raised after FFA treatment in AML-12 cells

**Figure 3:**
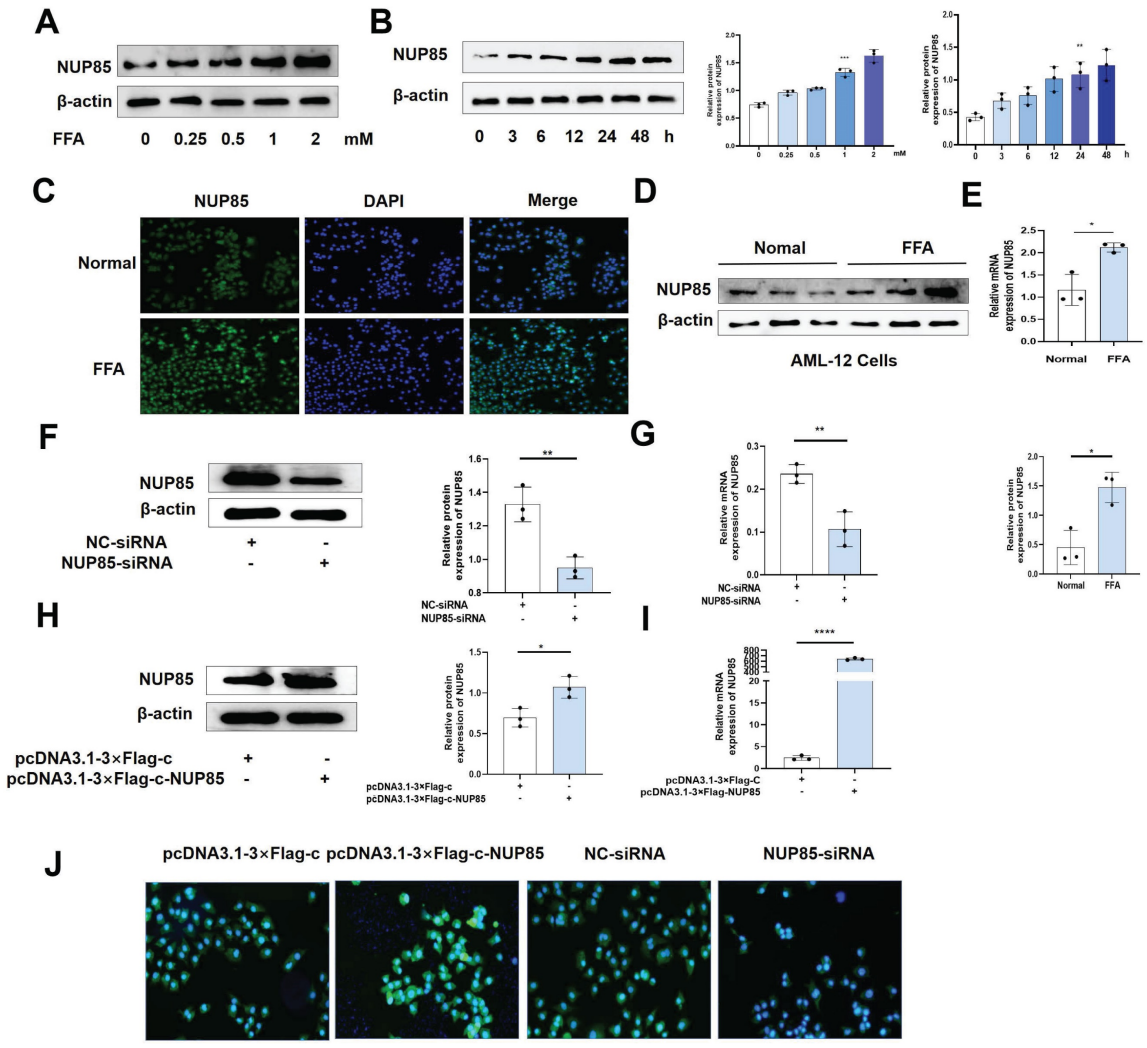
** The expression level of NUP85 is raised after FFA treatment in AML-12 cells.** A. The expression level of NUP85 protein with different FFA concentrations was detected by Western blotting. B. The expression level of NUP85 protein after 2 mm FFA treatment for 24 h was detected by Western blotting. C-E. NUP85 expression levels were detected by immunofluorescence, Western blotting and RT-qPCR. F-G. RT-qPCR and Western blotting were used to examine the expression level of NUP85 after transfected with NUP85-siRNA. H-I. RT-qPCR and Western blotting were used to examine the level of NUP85 after transfected with pcDNA3.1-3×Flag-c-NUP85. J. Immunofluorescence analysis was used to examine the level of NUP85 after transfected with pcDNA3.1-3×Flag-c-NUP85 and NUP85-siRNA. Measurement metrics are shown in the figure. All experimental results of this study were replicated at least three times. * *p<0.01, ***p<0.001 compared with the pair group.

To investigate the alteration of NUP85 expression in AML-12 cells, we tested the characteristics of NUP85 expression in FFA-treated AML-12 cells at different concentrations and times. Firstly, the results of CCK8 showed that the cell activity decreased in a FFA dose-dependent manner (Figure S2A). Secondly, the protein expression level of NUP85 reached the highest level with 2 mm FFA stimulation (Figure 3A). When AML-12 cells were stimulated with 2 mm FFA, NUP85 protein expression levels reached its peak at 24 h (Figure 3B). The results of Oil red O staining showed that FFA stimulation obviously aggravated the lipid accumulation in AML-12 cells (Figure S2B).* In vitro* models were determined to be stimulated with 2mm FFA for 24 h. Compared with the normal group, the expression level of NUP85 in the AML-12 cell treated with FFA was up-regulated (Figure 3C-3E). Next, we verified the transfection effect of NUP85. NUP85-siRNA and pcDNA3.1-3×Flag-c-NUP85 were used to transfect into AML-12 cells, and the relevant results indicated that NUP85 were successfully silenced and over-expressed (Figure 3F-3J). Hence, AML-12 cells were treated with 2 mm FFA for 24 h to detect NUP85 expression. The results showed the expression level of NUP85 is raised after FFA treatment in AML-12 cells.

### Interference with NUP85 mitigates lipid accumulation, inflammation and apoptosis in FFA-induced AML-12 cells

**Figure 4:**
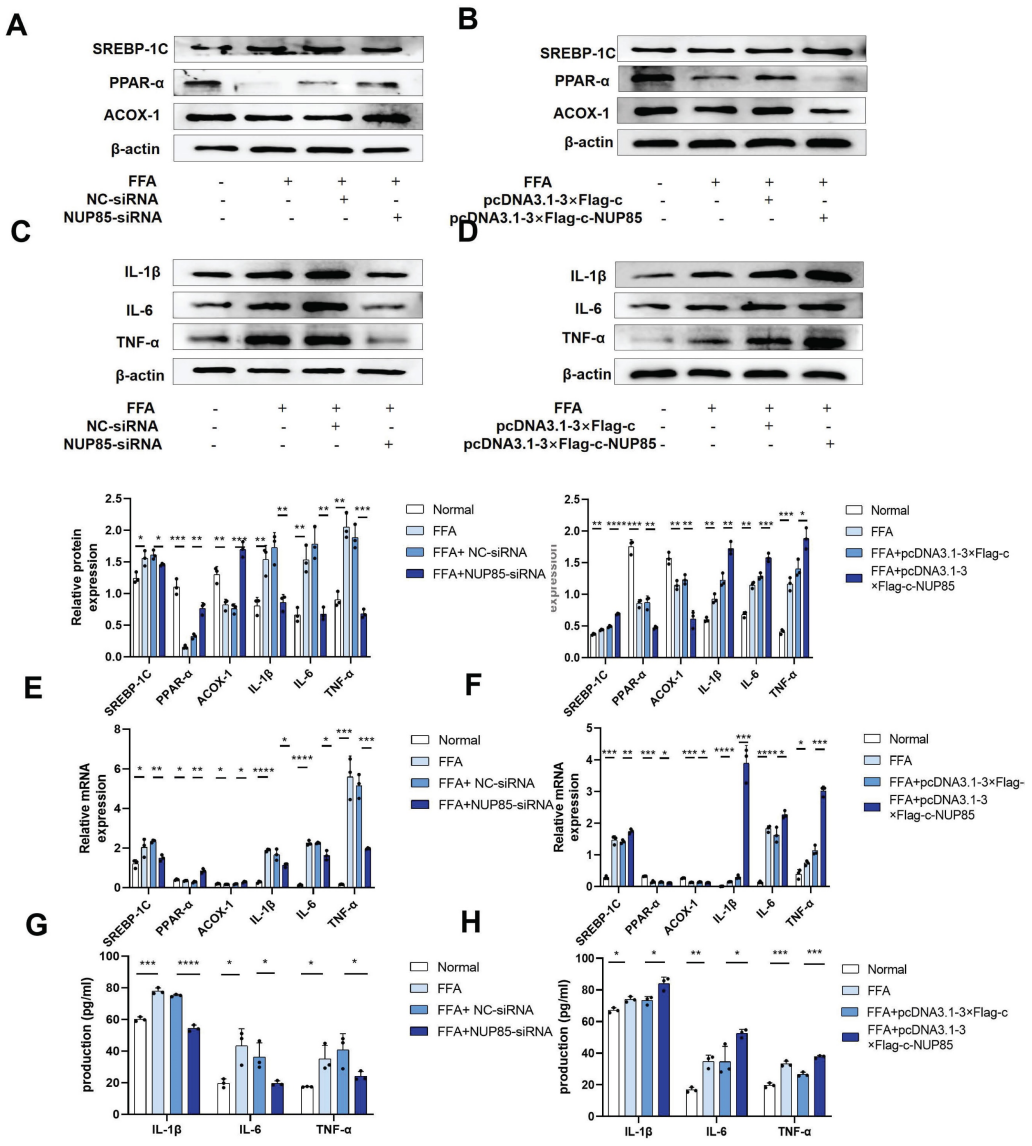
** Interference with NUP85 mitigated lipid accumulation, inflammation and apoptosis in FFA-induced AML-12 cells.** A-B and E. Western blotting and RT-qPCR were used to detect the protein and mRNA expression levels of SREBP-1C, PPAR-α and ACOX-1 in AML-12 cells transfected with NUP85-siRNA and pcDNA3.1-3×Flag-c-NUP85. C-D and F. After transfected with NUP85-siRNA and pcDNA3.1-3×Flag-c-NUP85, Western blotting and RT-qPCR were used to detect the protein and mRNA expression levels of IL-6, IL-1β and TNF-α. G-H Secretion levels of IL-1β, IL-6 and TNF-α in culture medium. All experimental results of this study were replicated at least three times. * *p<0.01, ***p<0.001 compared with the pair group.

To further investigate the role of NUP85 in lipid accumulation and inflammation, cell model was constructed. In NUP85-siRNA-transfected AML-12 cells, the expression levels of ACOX-1 and PPAR-α were increased, while the expression level of SREBP-1C was inhibited (Figure 4A and 4E). In addition, the expression levels of inflammation such as IL-1β, IL-6 and TNF-α were decreased in NUP85-siRNA-transfected AML-12 cells (Figure 4C and 4E). In order to investigate the influence of NUP85 over-expression on lipid accumulation and inflammation, pcDNA3.1-3×Flag-c-NUP85 was transfected into AML-12 cells. The results showed that the expression levels of PPAR-α and ACOX-1 were suppressed in pcDNA3.1-3×Flag-c-NUP85-transfected AML-12 cells, whereas the expression level of SREBP-1C was increased (Figure 4B and 4F). Additionally, the mRNA and protein expression levels of IL-1β, IL-6, and TNF-α were increased in pcDNA3.1-3×Flag-c-NUP85-transfected AML-12 cells (Figure 4D and 4F). Besides, the secretion of IL-1β, IL-6 and TNF-α were decreased in NUP85-siRNA-transfected AML-12 cells (Figure 4G). On the contrary, the secretion of IL-1β, IL-6 and TNF-α were increased in pcDNA3.1-3×Flag-c-NUP85-transfected AML-12 cells (Figure 4H). To further verify the effect of NUP85 on apoptosis flow cytometry (FCM) was performed firstly. Compared with the normal group, the apoptosis rate of FFA-stimulated model group was significantly increased. In addition, inhibition of NUP85 obviously reduced the apoptosis of liver cells. Oppositely, the apoptosis rate increased in NUP85 over-expression group (Figure S3A). Together, our findings implied that inhibiting NUP85 could lessen lipid accumulate, inflammation and apoptosis in AML-12 cells treated with FFA.

### NUP85 interacts with CCR2 in AML-12 cells

**Figure 5:**
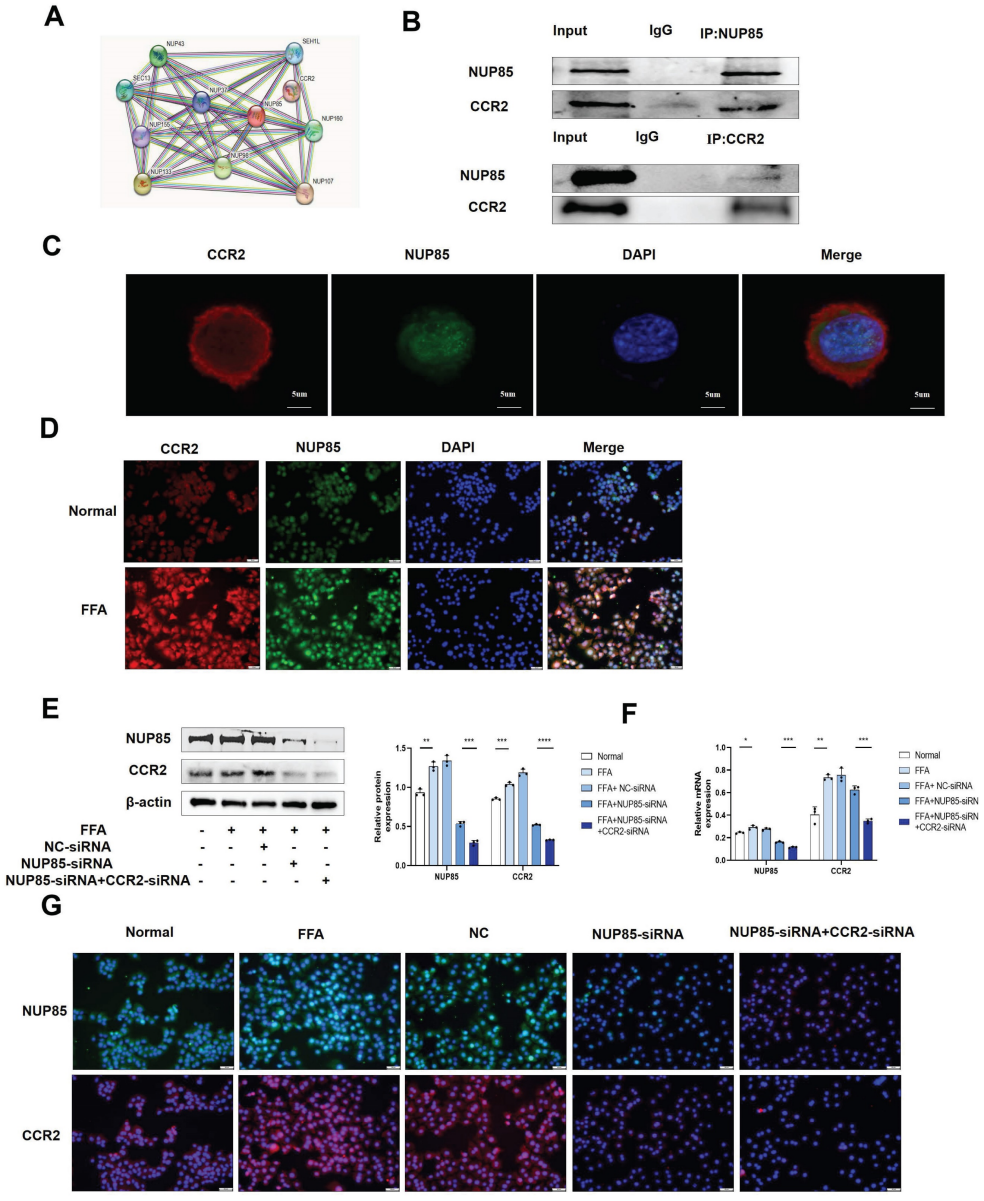
** NUP85 interacts with CCR2 in AML-12 cells.** A. The result of STRING database. B. CO-IP results showed that NUP85 combined with CCR2. C. Immunofluorescence staining bespeak the colocalization of NUP85 and CCR2. D. immunofluorescence analysis of CCR2. E-G. Western blotting, RT-qPCR and Immunofluorescence results of the expression levels of NUP85 and CCR2 in cells after transfected with NUP85-siRNA. Measurement metrics are shown in the figure. All experimental results of this study were replicated at least three times. * *p<0.01, ***p<0.001 compared with the pair group.

Next, we studied the regulatory mechanism of NUP85 in lipid accumulation and inflammation. In the STING database, NUP85 could bind to CCR2 (Figure 5A). Additionally, the expression level of CCR2 was obviously increased in the livers of the NAFLD patients, as IHC staining showed (Figure S4A). Similarly, IHC staining and Western blotting analysis showed higher CCR2 levels in the liver of MCD-fed mice compared to the MCS group (Figure S4B and S4C). Then, the CO-IP results (Figure 5B) and immunofluorescent staining results (Figure 5C) also confirmed that NUP85 bound to CCR2 in AML-12 cells. Additionally, the results of immunofluorescent staining and Western blotting showed higher levels of CCR2 in the FFA group (Figure 5D and S4D).

Furthermore, Western blotting and RT-qPCR results indicated that inhibition of NUP85 down-regulated the expression level of CCR2 in FFA-treated AML-12 cells, while NUP85-overexpression up-regulated the expression level of CCR2 (Figure S4E-S4G). To sum up, NUP85 could interact with CCR2 in AML-12 cells. To further verify the specific function of NUP85 combined with CCR2, NUP85-siRNA and CCR2-siRNA were co-transfected into FFA-induced AML-12 cells. Western blotting and RT-qPCR experiments showed the transfection was successful (Figure 5E-5F). The immunofluorescence results indicated that the expression levels of NUP85 and CCR2 in the co-transfection group were lower than that in the group transfected with NUP85-siRNA (Figure 5G). Besides, the co-transfection group manifested lower expression levels of SREBP-1C, IL-1β, IL-6 and TNF-α than the group transfected with NUP85-siRNA. Adversely, the co-transfection group showed higher expression levels of PPAR-α and ACOX-1 (Figure S4H-S4I). Thus, these results indicated that NUP85 could regulated lipid accumulation and inflammation by combining with CCR2 in AML-12 cells.

### NUP85 disruption attenuates lipid accumulation and inflammation in FFA-treated AML-12 cells by inhibiting the PI3K/AKT signaling pathway

**Figure 6:**
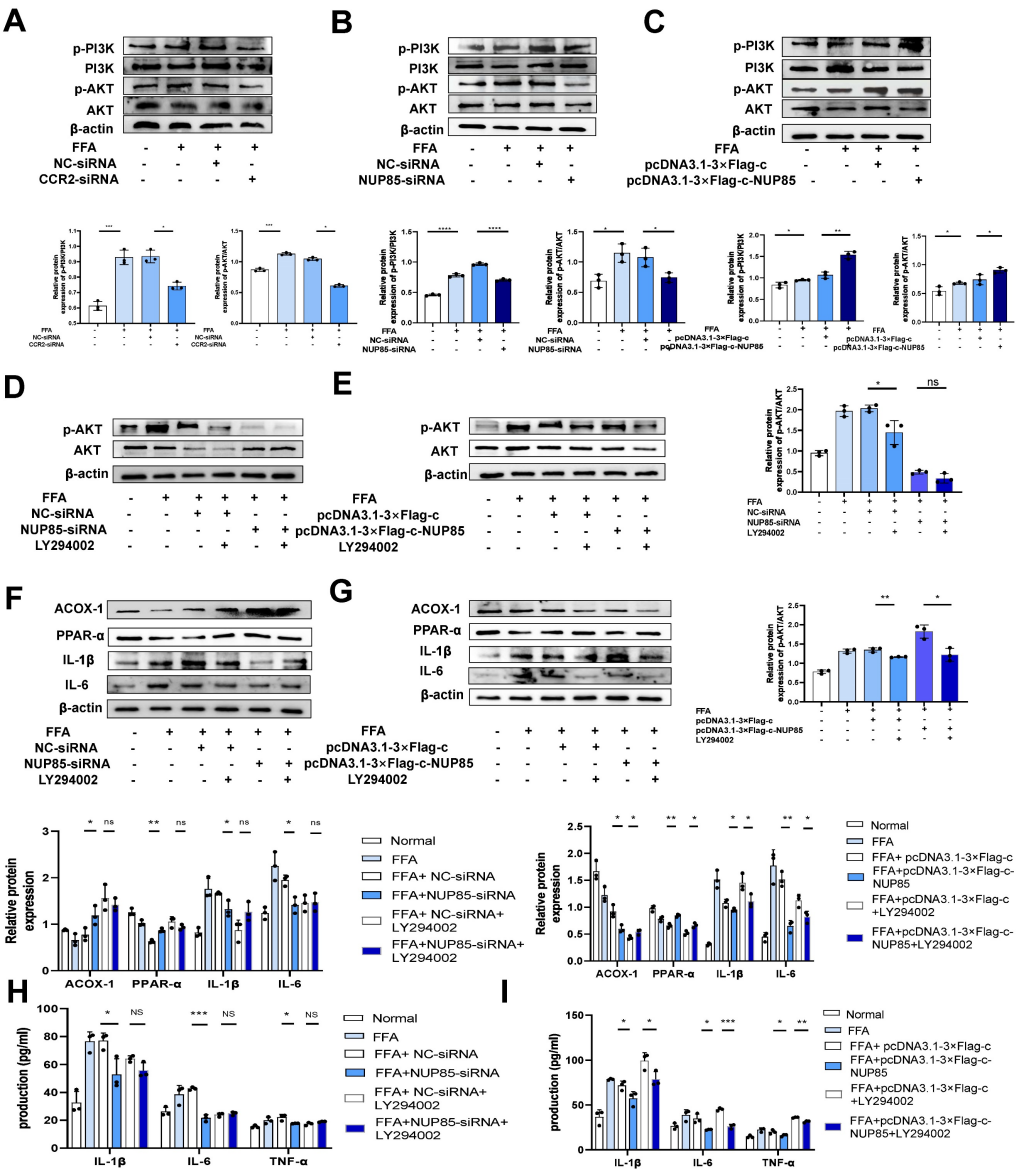
** NUP85 disruption attenuates lipid accumulation and inflammation in FFA-treated AML-12 cells by inhibiting the PI3K/AKT signaling pathway.** A. Western blotting was used to detect the expression levels of PI3K and p-PI3K in AML-12 cells after transfected with CCR2-siRNA. B-C. Western blotting results of the proteins associated with PI3K/AKT signaling pathways were tested in AML-12 cells after transfected with NUP85-siRNA and pcDNA3.1-3×Flag-c-NUP85. D-E. Western blotting was used to detect the expression levels of related proteins in the PI3K/AKT signaling pathway after transfected and co-cultured with LY294002. F-G. The protein expression levels of ACOX-1, PPAR-α, IL-6 and IL-1β were detected using Western blotting after transfected with NUP85-siRNA and pcDNA3.1-3× Flag-c-NUP85 and co-cultured with LY294002 for 8 h in AML-12 cells. H-I Secretion levels of IL-1β, IL-6 and TNF-α in culture medium. All experimental results of this study were replicated at least three times. **p<0.01, ***p<0.001 compared with the control group.

In order to further explore the regulatory mechanism of NUP85 on the PI3K/AKT signaling pathway in NAFLD. CCR2-siRNA was transfected into FFA-induced AML-12 cells revealed obviously lower levels of p-PI3K and p-AKT than the group transfected with NC-siRNA (Figure 6A). Besides, NUP85-siRNA and CCR2-siRNA were co-transfected into FFA-induced AML-12 cells revealed obviously lower levels of p-PI3K and p-AKT than the group transfected with NUP85-siRNA (Figure S5A). Western blotting was used to detect the expression levels of target proteins associated with the PI3K/AKT signaling pathway. Results indicated that NUP85-knockdown greatly down-regulated the expression of p-PI3K and p-AKT (Figure 6B). On the contrary, the expression level of p-PI3K and p-AKT were significantly increased after NUP85 overexpression (Figure 6C). The chemical structure of LY294002, a PI3K/AKT signaling pathway inhibitor, is shown in Figure S5B. To detect the effect of LY294002 on the viability of AML-12 cells, CCK-8 method was used. Different concentrations of LY294002 were applied to stimulate AML-12 cells for 8 h. With the increase of LY294002 concentration, cell viability gradually declined (Figure S5C). FFA-treated AML-12 cells were then co-cultured with LY294002 for 8 h. The result showed that LY294002 greatly suppressed the PI3K/AKT signaling pathway (Figure 6D-6E). Next, the expression levels of lipid metabolism and inflammatory cytokines-relevant proteins were detected. Western blotting results revealed that the expression levels of ACOX-1 and PPAR-α were significantly increased in NC-siRNA+LY294002 group, whereas IL-1β and IL-6 were tremendously decreased compared with NC-siRNA group. There were no discernible differences in the expression levels of ACOX-1, PPAR-α, IL-1β and IL-6 in the NUP85-siRNA+LY294002 group compared to the NUP85-siRNA group (Figure 6F). In contrast, ACOX-1 and PPAR-α expression levels in the pcDNA3.1-3×Flag-c+LY294002 group were significantly higher than those in the pcDNA3.1-3×Flag-c group, while IL-1β and IL-6 expression levels were lower. The expression levels of ACOX-1 and PPAR-α greatly raised in the pcDNA3.1-3×Flag-c-NUP85+LY294002 group compared to the pcDNA3.1-3×Flag-c-NUP85 group, while the expression level of IL-6 and IL-1β considerably declined (Figure 6G). Additionally, the secretion of inflammatory cytokines (IL-1β, IL-6 and TNF-α) confirmed that NUP85 can regulated inflammation through inhabiting the PI3K/AKT signaling pathway (Figure 6H-6I). In a word, NUP85 disruption attenuates lipid accumulation and inflammation in FFA-treated AML-12 cells by inhibiting the PI3K/AKT signaling pathway.

### NUP85 knockdown mitigates liver injury in MCD-fed mice

**Figure 7:**
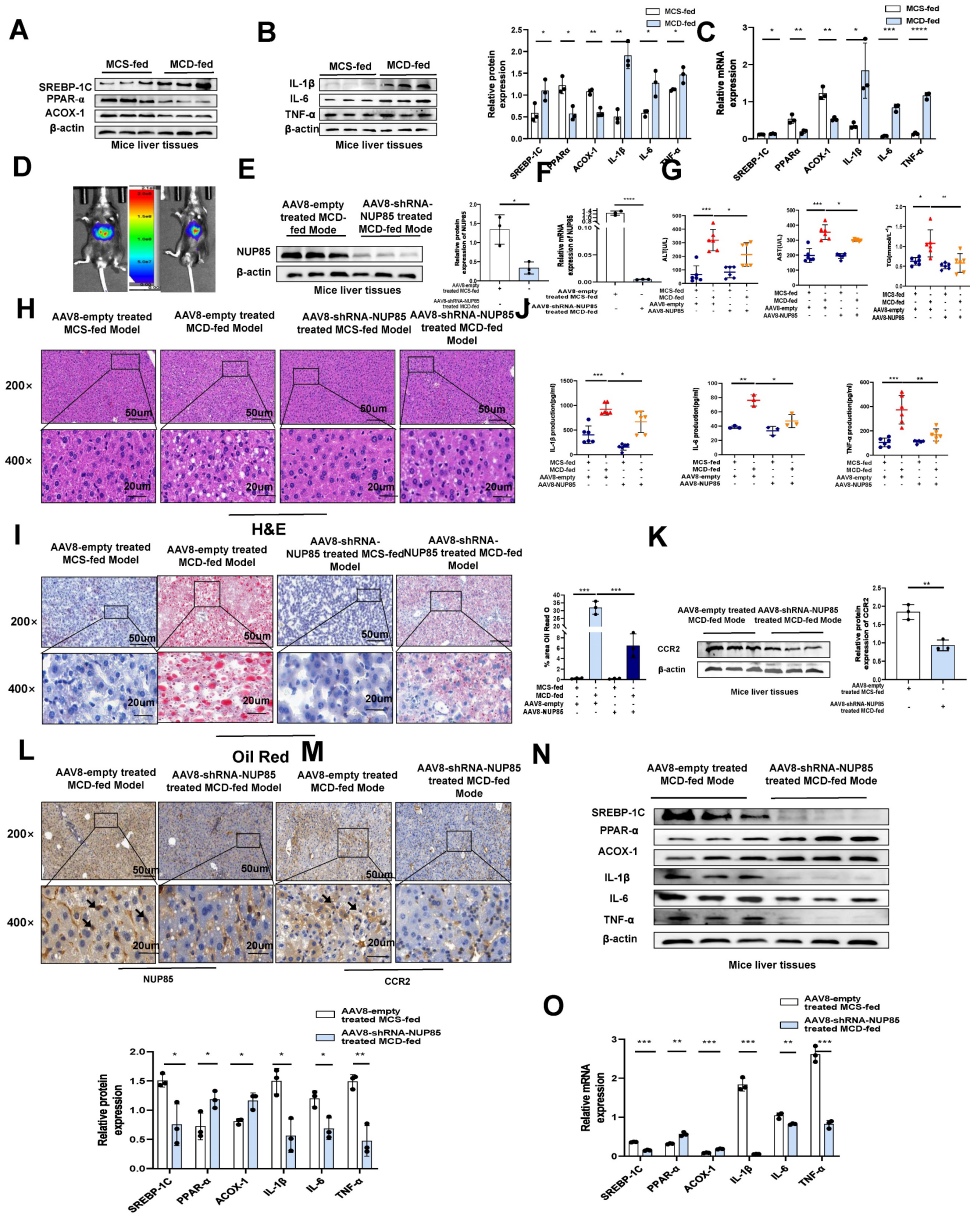
** NUP85 knockdown attenuates liver injury in MCD-fed mice.** A and C. The mRNA and protein expression levels of SREBP-1C, ACOX-1 and PPAR-α in mice liver tissues were detected by RT-qPCR and Western blotting. B and C. RT-qPCR and Western blotting were used to detect the expression levels of IL-6, TNF-α and IL-1β in the liver tissues of mice. D. Analysis of small animals imaging. E-F. The mRNA and protein expression levels of NUP85. G. Serum ALT, AST and TG assay. H. HE staining of liver tissues. I. Oil Red O staining in liver tissues. J. Levels of serum IL-1β, IL-6 and TNF-α. K. Western blotting results of the expression levels of CCR2. L-M. IHC analysis of NUP85 and CCR2. N-O. The mRNA and protein expression levels of IL-1β, IL-6, TNF-α, SREBP-1C, PPAR-α and ACOX-1. Measurement metrics are shown in the figure. All experimental results of this study were replicated at least three times. **p<0.01, ***p<0.001 compared with the control group.

To further verify the specific regulatory mechanism of NUP85 in alleviating NAFLD, the expression levels of lipid metabolism-related genes and proteins in mice liver tissues were examined. It pointed out that SREBP-1C expression level was up-regulated in mice fed with MCD diet compared to fed with MCS diet, while PPAR-α and ACOX-1 expression levels were down-regulated (Figure 7A and 7C). The impact of inflammatory cytokines also were examined in MCD-induced mice. The findings suggested that in the MCD group, the mRNA and protein expression levels of IL-1β, IL-6 and TNF-α were elevated (Figure 7B and 7C)). To test the function of NUP85, NUP85 was silenced through injecting AAV8-shRNA-NUP85 into the tail vein of mice 27 (Figure 7D and S6A ). Compared with the AAV8-NC group, the color the liver in the AAV8-shRNA-NUP85 group were more bright red (Figure S6B). There was no significant difference in body weight between the AAV8-NC group mice and the mice injected with AAV8-shRNA-NUP85 (Figure S6C-S6D). Analyses proved that the expression level of NUP85 was down-regulated in the AAV8-shRNA-NUP85 group (Figure 7E-7F). Compared with AAV8-NC group, the serum levels of ALT, AST and TG were decreased in AAV8-shRNA-NUP85 group (Figure 7G). Similarly, NUP85 knockdown had a liver protective effect on AAV8-shRNA-NUP85 group mice (Figure 7H).

Additionally, Oil Red O staining showed that the liver steatosis in AAV8-NC group was more serious (Figure 7I). Compared with AAV8-NC group, the levels of IL-1β, IL-6, and TNF-α in serum were significantly decreased in the AAV8-shRNA-NUP85 group (Figure 7J). TUNEL staining showed that NUP85 knockdown led to a decrease in the number of apoptotic liver cells compared to the AAV8-NC group (Figure S6E). It was certified that NUP85 could regulate CCR2 protein through the results of Western blotting analysis (Figure 7K). In conclusion, NUP85 knockdown could attenuate liver injury in MCD-fed mice. In the NUP85 knockdown group, the results of IHC analysis indicated that the expression levels of NUP85 and CCR2 were decreased (Figure 7L and 7M). According to our results, we also found lower expression levels of IL-6, IL-1β, TNF-α and SREBP-1C and higher expression levels of PPAR-α and ACOX-1 in AAV8-shRNA-NUP85 group (Figure 7N-7O). All the above results implied that NUP85 might be an effective target for preventing MCD diet-induced liver injury.

### ISRIB could target NUP85 to protect NAFLD

**Figure 8:**
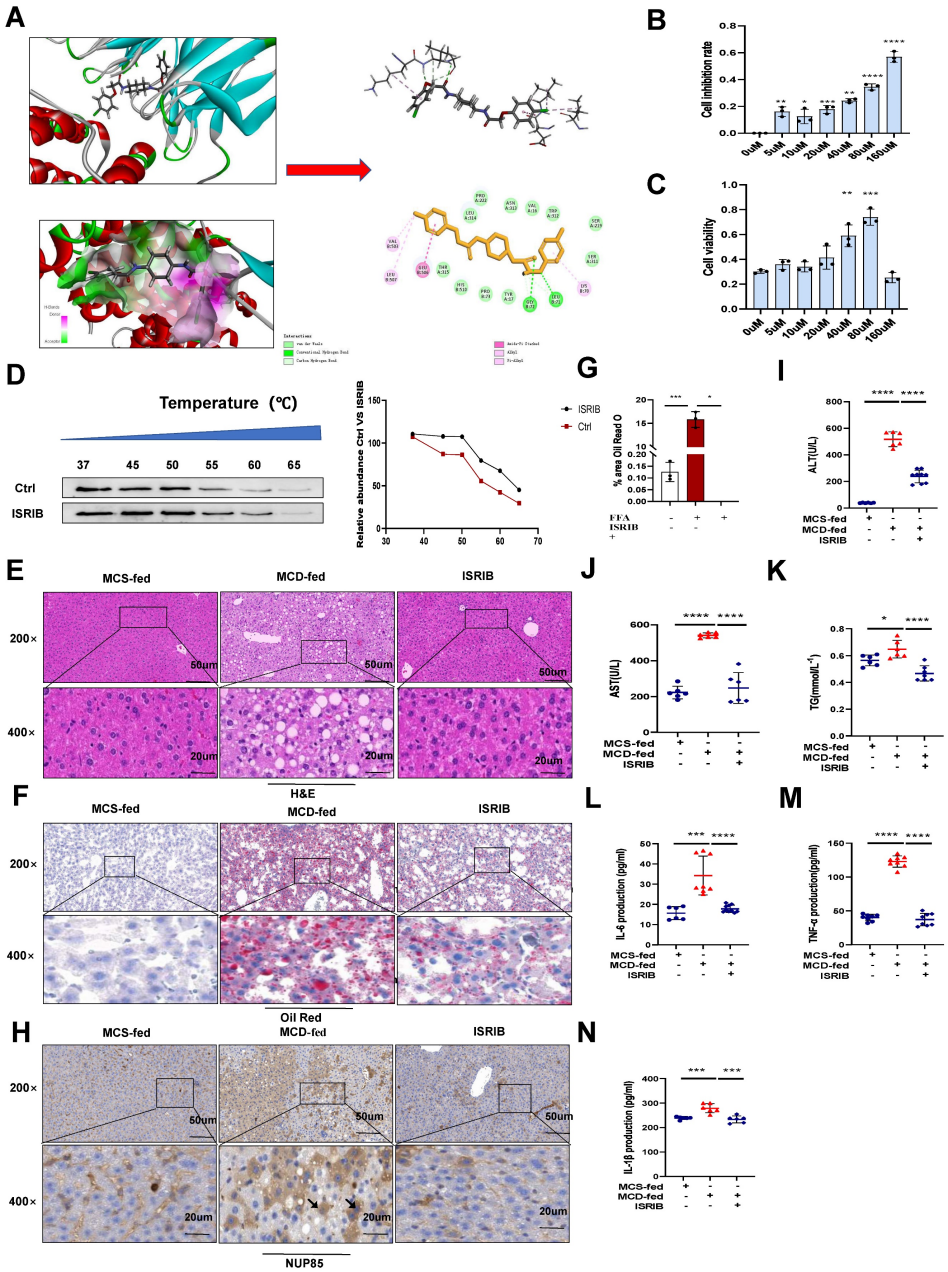
** ISRIB could target NUP85 to protect NAFLD.** A. Binding site of ISRIB to NUP85. B-C. *In vitro*, CCK8 was used to explore the concentration of ISRIB. D. AML-12 cells were cultivated with or without ISRIB (80 um) for 24 h and CETSA test was performed. E-G. H&E staining and Oil Red O staining of liver sections. H. Immunohistochemistry of liver tissues. I-K. Serum ALT, AST and TG assay. L-N. Levels of serum IL-1β, IL-6 and TNF-α. Measurement metrics are shown in the figure. All experimental results of this study were replicated at least three times. **p<0.01, ***p<0.001 compared with the control group.

Based on the above results, the experimental results showed that NUP85 could alleviate NAFLD. Therefore, it might be important to find the effective drug targets for NUP85. Thus, our study focused on seeking target drugs of NUP85 for the treatment of NAFLD. In this study, we attempted to use a virtual screening approach to find a new inhibitor targeting NUP85 from classic drugs and inhibitors which were already on the market 28. Luckily, ISRIB has been found to be able to inhibit NUP85. Molecular docking studies 29 were designed to further understand the binding pattern and direction of the target compound to the NUP85 binding site. The study was used to assess how the small molecules fit with the target macromolecule (receptor). In Discovery Studio2018 software, the C-Docker protocol was used for docking research. The C-Docker algorithm was used to re-interface the NUP85 active site and verify the docking protocol. After the molecular docking, the types of interactions between the docking proteins and ligands were analyzed. The carbonyl and oxygen atoms in one end of the molecule form hydrogen bond with GLY72 and LEU71, respectively. In addition, at the other end of the molecule, we could find that the benzene ring forms Amide-Pi stacked interactions with GLU506, and the chlorine atoms form Pi-Alkyl interactions with VAL503 and LEU507. Docking results of the compound could be seen in (Figure 8A). In order to explore the pharmacological effects of ISRIB, CCK8 experiment was used, and the results indicated that 80 μm ISRIB had a protective function on AML-12 cells stimulated by FFA (Figure 8B and 8C). In subsequent experiments, the efficacy of ISRIB *in vitro* was deeply explored at the concentration of 80 μm. In addition, CETSA was used to prove the combination between ISRIB and NUP85 (Figure 8D). According to the principle, when the drug bound to the protein, the stability of the NUP85 protein increased. As shown in Figure 8D, NUP85 almost disappeared at 55 ℃ in ctrl group, while NUP85 disappeared at 60 ℃ in cells treated with ISRIB. We next explored the therapeutic potential of targeting NUP85 during the development of NAFLD *in vivo*. H&E and Oil Red O staining showed that liver cells ballooning and lipid droplets were reduced in the MCD group of mice treated with ISRIB (Figure 8E-8G). Immunohistochemical results showed that the expression level of NUP85 was decreased in ISRIB group (Figure 8H). In addition, compared with the MCD group, the levels of ALT, AST and TG were also significantly reduced in the ISRIB group (Figure 8I-8K). The results of ELISA showed that the expression levels of IL-1β, IL-6 and TNF-α were decreased in ISRIB group (Figure 8L-8N). All in all, ISRIB could target NUP85 to protect NAFLD.

## Discussion

NAFLD is a chronic liver disease characterized by diffuse liver cell steatosis 30, 31. NAFLD date from NAFL, NASH, gradually worsens to related cirrhosis and finally develops into HCC 32, 33. Further research shows that lipid accumulation, inflammation and other factors can aggravate and amplify the lipid metabolism and inflammation of the liver 34. It is worth noting that the inhibition of steatosis and inflammation have become the key to prevent the gradual deterioration of NAFLD 35. MCD group mice with severe hepatic steatosis and obvious inflammation had similar pathological characteristics to those of human NAFLD except for no weight gain 36, 37. Besides, MCD diet-induced fatty liver led to a large number of fat vacuoles, and the levels of serum ALT, AST and TG in MCD group significantly increased compared with those in MCS group. *In vitro*, FFA treatment induced lower expression levels of PPAR-α and ACOX-1 and higher expression levels of SREBP-1C, IL-1β, IL-6 and TNF-α. TUNEL staining also showed that the apoptosis rate distinctly increased in the MCD group. Thus, MCD diet induced lipid accumulation and inflammation in C57BL/6J mice.

NUP85 regulated macrophages to affect liver inflammation 38, and NUP85 was very important in the progress of tumor genesis 39. However, the molecular mechanism of up-regulation of NUP85 expression in NAFLD was unclear.

Based on the above results, we transfected cells with NUP85-siRNA and constructed NUP85-knockdown mice model whether NUP85 affected MCD-induced NAFLD. In addition, NUP85 played a key regulatory role in various processes, including stress and inflammation 38, 39. Importantly, Kulyte A et al. found that NUP85 was an intrinsic regulator of lipolysis and the association was consistent in the clinical cohort 40. Lipid accumulation and inflammation were very essential for the germination and progress of NAFLD 41, 42. In this work, the expression levels of lipogenic and inflammatory factor-related proteins were inhibited in NUP85-siRNA-transfected AML-12 cells. In addition, knockdown of NUP85 could alleviate inflammation and lipid accumulation in MCD-induced liver injury.

Macrophage autophagy mediated by partner loss aggravates inflammation of NASH by targeting NUP85 43. NUP85 could combine with CCR2 protein by querying STING database 44. CCR2 is the chemokine receptor, which regulates the mobilization of monocytes from bone marrow to the inflammatory sites 45,46. Interestingly, the circulating precursor of macrophages could control the migration of bone marrow monocytes to the liver 47. Puengel T et al. found that combined treatment with CCR2/CCR5 antagonists and FGF21 analogs synergistically improved steatohepatitis and fibrosis 48. Yuya Terashima et al. confirmed that NUP85 could bind to CCR2 49, significantly promoting tumor progression and macrophage pro-tumor activity 39. It had been documented that it could regulate the inflammation and lipid accumulation in some organs of the body 50. In this study, the results explained that NUP85 could bind to CCR2 and increased the expression level of CCR2 protein in AML-12 cells induced by FFA, thereby aggravating MCD-induced NAFLD. Furthermore, the study showed that NUP85 interacted with CCR2 to regulate the PI3K/AKT signaling pathway, thus affecting lipid accumulation and inflammation in NAFLD. ISRIB, a targeted inhibition of NUP85, alleviated NAFLD. To summarize, it was suggested that NUP85 pharmacological inhibition might provide a feasible treatment for MCD-induced liver injury.

Despite the new findings reported here, potential limitations of this study should be noted. Firstly, we injected AAV8-shRNA-NUP85 through the tail vein, and the results of Western blotting and RT-qPCR showed that the expression level of NUP85 in mouse liver tissue was reduced. However, compared with NUP85 knockout mice, this method has some limitations in the variable infection rate and efficiency of NUP85 knockout. Secondly, we only use MCD modeling method to establish NAFLD model. In the future, it is necessary to deeply explore the comprehensive mechanisms of NUP85 by constructing NUP85 knockout mice and using other modeling methods such as HFD and Western diet.

Functionally, NUP85 can alleviate lipid accumulation and inflammation, which provides a potential rationale for clinical drugs studies about targeting NUP85 with ISRIB could treat NAFLD. Mechanically, we found a new mechanism that NUP85 links activated PI3K/AKT signaling pathway, which can affect NAFLD. This has also been demonstrated by LY294002 inhibiting the PI3K/AKT signaling pathway, thereby reducing lipid accumulation and inflammation in NAFLD. Future, various sophisticated biology tools will be used to investigate the molecular mechanisms of NUP85 in NAFLD, which can develop promising treatment strategies.

## Supplementary Material

Supplementary figures and table.

## Abbreviations

NAFLD: nonalcoholic fatty liver disease
TG: triglyceride
NUP85: nucleoporin 85
MCD: methionine-choline-deficient
FFA: free fatty acids
CCR2: C-C motif chemokine receptor 2
PI3K: phosphoinositol-3 kinase
AKT: protein kinase B
ISRIB: trans isomer
NAFL: nonalcoholic fatty liver
NASH: nonalcoholic steatohepatitis
NPC: Nuclear pore complexes
NUPs: nucleoporins
HCC: hepatocellular carcinoma
ACOX-1: acyl-CoAoxidase-1
IL-1β: interleukin-1beta
SREBP-1C: sterol regulatory element binding protein-1
PPAR-α: peroxisome proliferator-activated receptor alpha
TNF-α: tumor necrosis factor-alpha
IL-6: interleukin-6
ALT: Alanine aminotransferase
AST: aspartate aminotransferase
USTC: the University of Science and Technology of China
MCS: methionine-choline supplementation
AAV: adeno-associated virus
Ip: intraperitoneally
OA: oleic acid
PA: palmitic acid
IHC: Immunohistochemistry
IF: Immunofluorescence
HE: Haematoxylin eosin
CO-IP: Co Immunoprecipitation
CETSA: Cellular thermal shift assay
BMl: body mass index
TC: total cholesterol
HDL-C: high density lipoprotein-cholesterol
