# Supplementary material for: NUP85 alleviates lipid metabolism and inflammation by regulating PI3K/AKT signaling pathway in nonalcoholic fatty liver disease
Source: Int J Biol Sci. 2024 Mar 25;20(6):2219–35. doi: 10.7150/ijbs.92337 (PMC11008257; doi:10.7150/ijbs.92337)
Supplement: Supplementary file 1 — Supplementary figures and table. [file ijbsv20p2219s1.pdf]

Supplemental materials

NUP85 alleviates lipid metabolism and inflammation by regulating PI3K/AKT signaling pathway in nonalcoholic fatty liver disease

Authors

Yin-cui Wu [a;b;1], Qi Yan [c;1], Si-qing Yue [a;b], Lin-xin Pan [d], Da-shuai Yang [a;b], Liang-song Tao [a;b], Ze-yuan Wei [a;b], Fan Rong [a;b], Cheng Qian [e], Meng-qi Han [a;b], Fu-cheng Zuo [a;b], Jun-fa Yang [a;b], Jia-jia Xu [a], Zheng-rong Shi [f], Jian Du [c*], Zhao-lin Chen [g, h*], Tao Xu [a;b*]

[a] Inflammation and Immune Mediated Diseases Laboratory of Anhui Province, Anhui Institute of Innovative Drugs, School of Pharmacy, Anhui Medical University, Hefei, 230032, China.

[b] Institute for Liver Diseases of Anhui Medical University.

[c] School of Basic Medical Sciences, Anhui Medical University, Hefei 230032, China.

[d] College of life sciences, Anhui Medical university.

[e] Research and Experiment center, Anhui Medical university.

[f] Department of Hepatobiliary Surgery, The First Affiliated Hospital of Chongqing Medical University, Chongqing, China.

[g] Department of Pharmacy, The First Affiliated Hospital of USTC, Division of Life Sciences and Medicine, University of Science and Technology of China, Anhui Provincial Hospital, Hefei, Anhui, 230001, P.R. China.

[h] Anhui Provincial Key Laboratory of Precision Pharmaceutical Preparations and Clinical Pharmacy, Hefei, Anhui, 230001, China.

[1]They contribute equally to this work.

[*] Corresponding author

Jian Du

School of Basic Medical Sciences

Anhui Medical University

Hefei, Anhui Province, 230032,

China.
E-mail: dujian@ahmu.edu.cn

Zhaolin Chen
Department of Pharmacy
The First Affiliated Hospital of USTC
Hefei, Anhui Province, 230032,
China.
E-mail: czl0808@ustc.edu.cn

Tao Xu
School of Pharmacy,
Anhui Medical University,
81 Meishan Road,
Hefei, Anhui Province, 230032,
China.
Tel/fax: +86 0551-65172131
E-mail: xutao@ahmu.edu.cn

Supplementary Table 1. The characteristics of NAFLD patients and healthy subjects.

	healthy subjects	NAFLD patients	*P value*
Age (years)	58±4.36	59.77±10.14	NS
BMI (kg/m^2)	25.6±3.78	23.42±3.24	NS
ALT (U/L)	19.33±11.15	40.16±20.36	<0.05
AST (U/L)	17.67±7.37	36.25±19.41	<0.05
TC (mmoI/L)	5.7±0.3	4.32±0.78	<0.05
TG (mmoI/L)	1.96±0.5	1.37±0.51	<0.05
HDL-C(mmol/L)	1.27±0.26	1.08±0.34	<0.05
Glucose(mmol/L)	5.28±0.47	6.25±1.6	<0.05

BMl: body mass index; ALT: alanine transaminase; AST: aspartate transaminase; TC: total cholesterol; HDL-C: high density lipoprotein-cholesterol.

Figure legends

Figure S1. A. Animal experimental design. B. Changes of body weight in mice. C. Weight of mice. D. Pictures of mice liver tissues. E. TUNEL staining of mice liver tissues. F. Detection of NUP85 and Albumin in liver by immunofluorescence staining. Measurement metrics are shown in the figure. All experimental results of this study were replicated at least three times. **p<0.01, ***p<0.001 compared with the pair group.

Figure S2. A. AML-12 cells were co-cultured with different concentration (0, 0.0625, 0.125, 0.25, 0.5, 1, 2, 4, 8 mm) of FFA for 24 h to detect cell viability. B. Oil red O staining. The percentage of lipid area in liver sections was detected by Oil red O staining. All experimental results of this study were replicated at least three times. **p<0.01, ***p<0.001 compared with the pair group.

Figure S3. A. Apoptosis of AML-12 cells was assessed using FCM. All experimental results of this study were replicated at least three times. * *p<0.01, ***p<0.001 compared with the pair group.

Figure S4. NUP85 silencing alleviates FFA-induced lipid disorders and inflammation in AML-12 cells by interacting with CCR2. A. IHC results analysis of CCR2 in NAFLD patients. B-C. IHC and Western blotting results of CCR2 in mice liver tissues. D. Western blotting analysis of CCR2. E-G. Western blotting and RT-qPCR results of the levels of CCR2 after transfected with NUP85-siRNA and pcDNA3.1-3×Flag-c-NUP85 in cells. H-I. Western blotting and RT-qPCR were used to detect the expression levels of SREBP-1C, IL-1β, IL-6 and TNF-α, PPAR-α and ACOX-1. All experimental results of this study were replicated at least three times. * *p<0.01, ***p<0.001 compared with the pair group.

Figure S5. A. Western blotting was used to detect the expression levels of PI3K and p-PI3K in AML-12 cells after transfected with CCR2-siRNA and NUP85-siRNA. B. Chemical structure of LY294002. C. CCK8 was used to detect cell activity. All experimental results of this study were replicated at least three times. * *p<0.01, ***p<0.001 compared with the pair group.

Figure S6. A. Analysis of small animals Imaging. B-D. Macroscopic appearance and body weight of the livers of mice. E. TUNEL staining of mice liver tissues.All experimental results of this study were replicated at least three times. * *p<0.01, ***p<0.001 compared with the pair group.

Figure S1

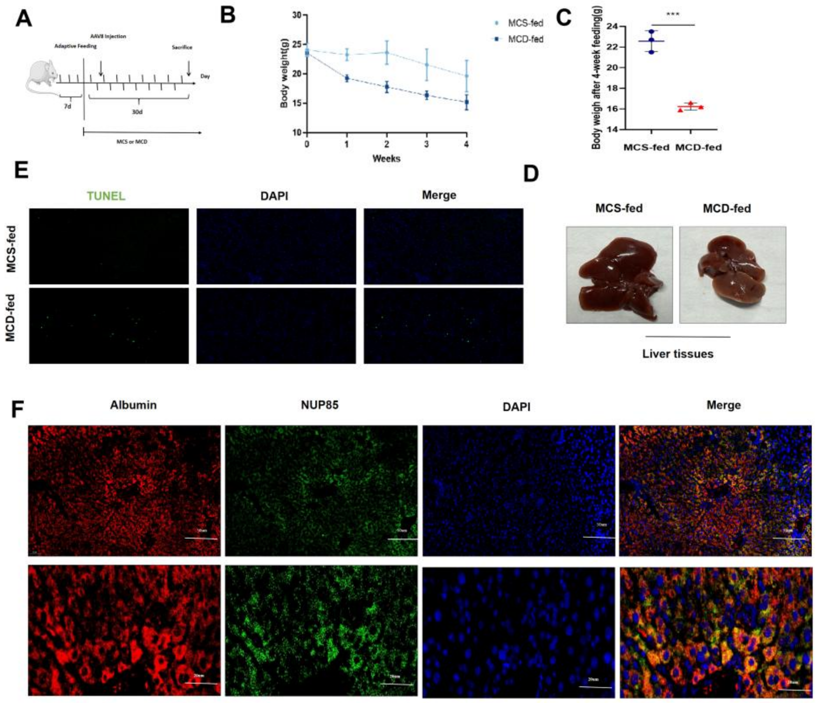
A
Adaptive Feeding
AAV8 Injection
Sacrifice
Day
7d
30d
MCS or MCD
B
Body weight(g)
Weeks
MCS-fed
MCD-fed
C
***
Body weigh after 4-week feeding(g)
MCS-fed
MCD-fed
D
MCS-fed
MCD-fed
Liver tissues
E
TUNEL
DAPI
Merge
MCS-fed
MCD-fed
F
Albumin
NUP85
DAPI
Merge

Figure S2

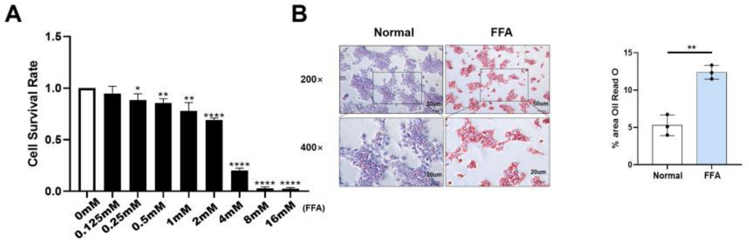
A
Cell Survival Rate
1.5
1.0
0.5
0.0
*
**
**
****
****
****
****
0mM
0.125mM
0.25mM
0.5mM
1mM
2mM
4mM
8mM
16mM
(FFA)
B
Normal
FFA
200×
400×
50um
20um
% area Oil Read O
15
10
5
0
**
Normal
FFA

Figure S3

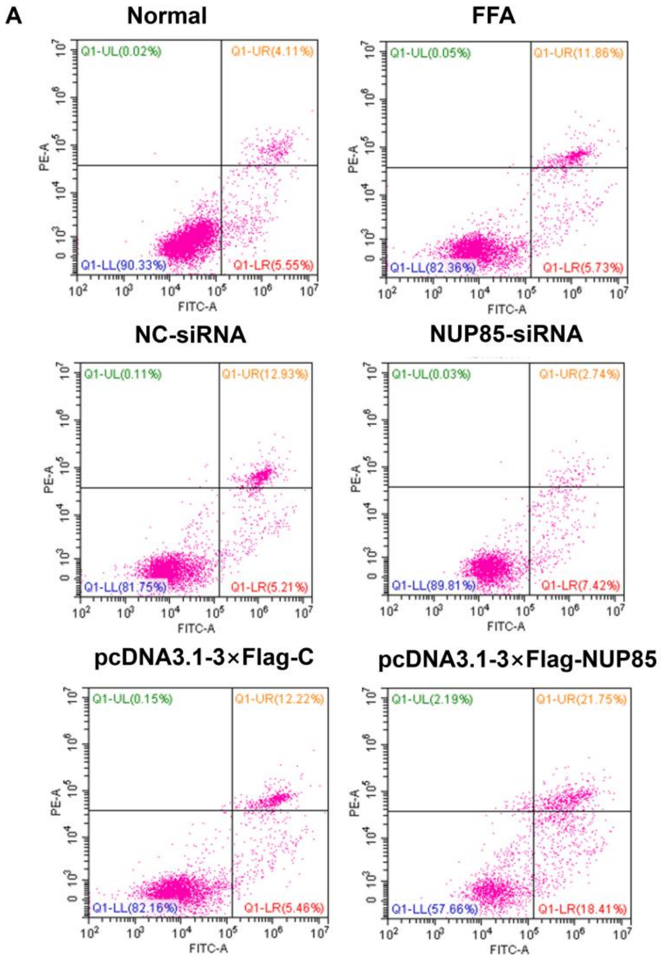
A
Normal
Q1-UL(0.02%)
Q1-UR(4.11%)
Q1-LL(90.33%)
Q1-LR(5.55%)
FFA
Q1-UL(0.05%)
Q1-UR(11.86%)
Q1-LL(82.36%)
Q1-LR(5.73%)
NC-siRNA
Q1-UL(0.11%)
Q1-UR(12.93%)
Q1-LL(81.75%)
Q1-LR(5.21%)
NUP85-siRNA
Q1-UL(0.03%)
Q1-UR(2.74%)
Q1-LL(89.81%)
Q1-LR(7.42%)
pcDNA3.1-3×Flag-C
Q1-UL(0.15%)
Q1-UR(12.22%)
Q1-LL(82.16%)
Q1-LR(5.46%)
pcDNA3.1-3×Flag-NUP85
Q1-UL(2.19%)
Q1-UR(21.75%)
Q1-LL(57.66%)
Q1-LR(18.41%)
PE-A
FITC-A

Figure S4

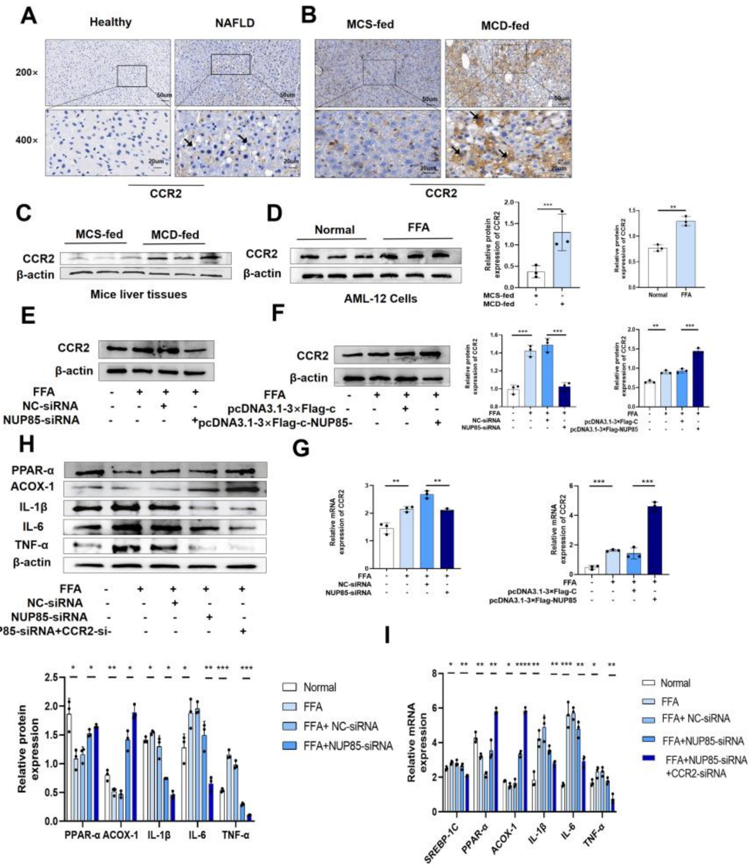
A
Healthy
NAFLD
200×
400×
CCR2
B
MCS-fed
MCD-fed
CCR2
C
MCS-fed
MCD-fed
CCR2
β-actin
Mice liver tissues
D
Normal
FFA
CCR2
β-actin
AML-12 Cells
E
CCR2
β-actin
FFA
NC-siRNA
NUP85-siRNA
F
CCR2
β-actin
FFA
pcDNA3.1-3×Flag-c
pcDNA3.1-3×Flag-c-NUP85-
G
H
PPAR-α
ACOX-1
IL-1β
IL-6
TNF-α
β-actin
FFA
NC-siRNA
NUP85-siRNA
NUP85-siRNA+CCR2-si-
Relative protein expression
Normal
FFA
FFA+ NC-siRNA
FFA+NUP85-siRNA
I
Relative mRNA expression
Normal
FFA
FFA+ NC-siRNA
FFA+NUP85-siRNA
FFA+NUP85-siRNA
+CCR2-siRNA

Figure S5

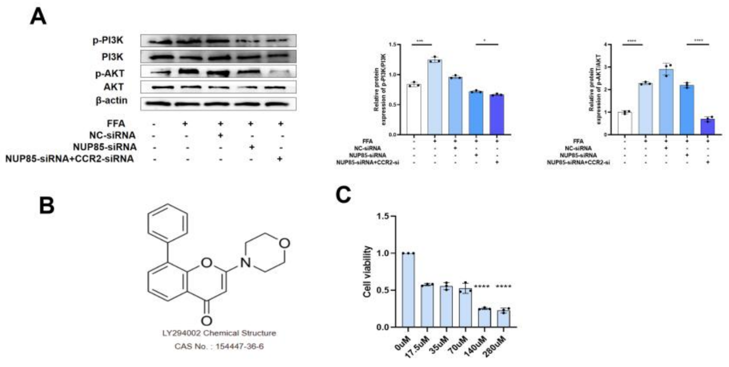

A
p-PI3K
PI3K
p-AKT
AKT
β-actin
FFA
NC-siRNA
NUP85-siRNA
NUP85-siRNA+CCR2-siRNA
B
LY294002 Chemical Structure
CAS No. : 154447-36-6
C
Cell viability
0uM
17.5uM
35uM
70uM
140uM
280uM

Figure S6

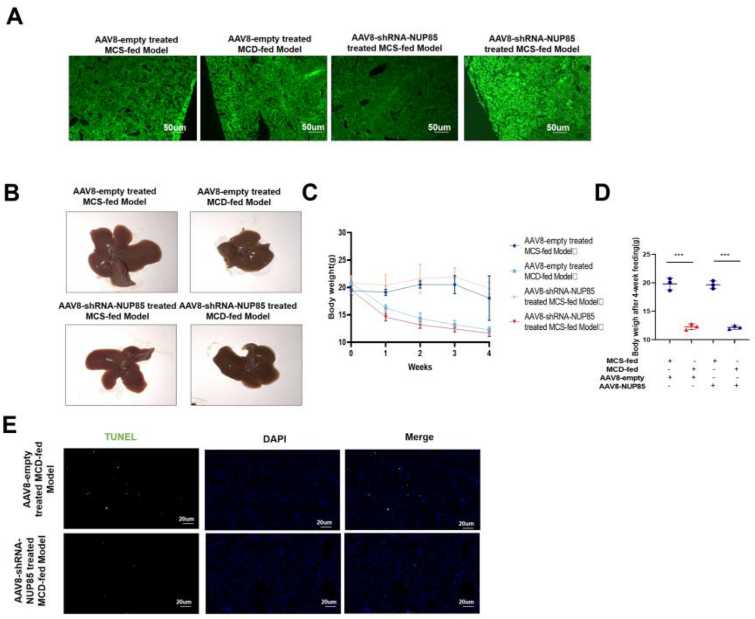
A
AAV8-empty treated MCS-fed Model
AAV8-empty treated MCD-fed Model
AAV8-shRNA-NUP85 treated MCS-fed Model
AAV8-shRNA-NUP85 treated MCS-fed Model
50um
B
AAV8-empty treated MCS-fed Model
AAV8-empty treated MCD-fed Model
AAV8-shRNA-NUP85 treated MCS-fed Model
AAV8-shRNA-NUP85 treated MCD-fed Model
C
Body weight(g)
Weeks
AAV8-empty treated MCS-fed Model
AAV8-empty treated MCD-fed Model
AAV8-shRNA-NUP85 treated MCS-fed Model
AAV8-shRNA-NUP85 treated MCS-fed Model
D
Body weigh after 4-week feeding(g)
***
MCS-fed
MCD-fed
AAV8-empty
AAV8-NUP85
E
TUNEL
DAPI
Merge
AAV8-empty treated MCD-fed Model
AAV8-shRNA-NUP85 treated MCD-fed Model
20um
